# What Factors Affect Outcome in the Treatment of Fracture-Related Infection?

**DOI:** 10.3390/antibiotics11070946

**Published:** 2022-07-14

**Authors:** Martin McNally, Ruth Corrigan, Jonathan Sliepen, Maria Dudareva, Rob Rentenaar, Frank IJpma, Bridget L. Atkins, Marjan Wouthuyzen-Bakker, Geertje Govaert

**Affiliations:** 1 Bone Infection Unit, Nuffield Orthopaedic Centre, Oxford University Hospitals, Oxford OX3 9DU, UK; ruth.corrigan@doctors.org.uk (R.C.); maria.dudareva@ouh.nhs.uk (M.D.); bridget.atkins@ouh.nhs.uk (B.L.A.); 2 Nuffield Department of Clinical Laboratory Sciences, Oxford University, John Radcliffe Hospital, Oxford OX3 9DU, UK; 3 Department of Trauma Surgery, University Medical Centre Groningen, University of Groningen, 9712 CP Groningen, The Netherlands; j.sliepen@umcg.nl (J.S.); frankijpma@gmail.com (F.I.); 4 Department of Medical Microbiology, University Medical Centre Utrecht, 3584 CX Utrecht, The Netherlands; rrentenaar@yahoo.com; 5 Department of Medical Microbiology and Infection Prevention, University Medical Centre Groningen, University of Groningen, 9712 CP Groningen, The Netherlands; m.wouthuyzen@icloud.com; 6 Department of Trauma Surgery, University Medical Centre Utrecht, 3584 CX Utrecht, The Netherlands; g.a.m.govaert@umcutrecht.nl

**Keywords:** fracture-related infection, non-union, infection, fracture, outcome, DAIR, timing, local antibiotics

## Abstract

This international, multi-center study investigated the effect of individual components of surgery on the clinical outcomes of patients treated for fracture-related infection (FRI). All patients with surgically treated FRIs, confirmed by the FRI consensus definition, were included. Data were collected on demographics, time from injury to FRI surgery, soft tissue reconstruction, stabilization and systemic and local anti-microbial therapy. Patients were followed up for a minimum of one year. In total, 433 patients were treated with a mean age of 49.7 years (17–84). The mean follow-up time was 26 months (range 12–72). The eradication of infection was successful in 86.4% of all cases and 86.0% of unhealed infected fractures were healed at the final review. In total, 3.3% required amputation. The outcome was not dependent on age, BMI, the presence of metalwork or time from injury (recurrence rate 16.5% in FRI treated at 1–10 weeks after injury; 13.1% at 11–52 weeks; 12.1% at >52 weeks: *p* = 0.52). The debridement and retention of a stable implant (DAIR) had a failure rate of 21.4%; implant exchange to a new internal fixation had a failure rate of 12.5%; and conversion to external fixation had a failure rate of 10.3% (adjusted hazard ratio (aHR) DAIR vs. Ext Fix 2.377; 95% C.I. 0.96–5.731). Tibial FRI treated with a free flap was successful in 92.1% of cases and in 80.4% of cases without a free flap (HR 0.38; 95% C.I. 0.14–1.0), while the use of NPWT was associated with higher recurrence rates (HR 3.473; 95% C.I. 1.852–6.512). The implantation of local antibiotics reduced the recurrence from 18.7% to 10.0% (HR 0.48; 95% C.I. 0.29–0.81). The successful treatment of FRI was multi-factorial. These data suggested that treatment decisions should not be based on time from injury alone, as other factors also affected the outcome. Further work to determine the best indications for DAIR, free flap reconstruction and local antibiotics is warranted.

## 1. Introduction

Good outcomes in fracture care can be adversely affected by fracture-related infection (FRI), leading to non-union and, occasionally, limb loss [1]. This presents a major burden for patients and healthcare systems around the world [2,3]. Direct hospital costs of FRIs are two to eight times higher than the costs for similar non-infected fractures, with a prolonged loss of employment and a requirement for welfare support [3,4]. FRIs occur in between 1 and 30% of fractures, depending on the injury severity and initial management [5,6].

Despite this large burden, treatment strategies have not been standardized and it is difficult to compare clinical trials. The introduction of an internationally endorsed consensus on the definition of FRI [7,8] may help to focus studies and allow for a better evaluation of treatments. The principles of treatments (diagnostic sampling, excision of dead tissue, stabilization, dead-space management, soft tissue cover and anti-microbial therapy) are well established [1,9], but the effectiveness of how each of these principles is delivered has not been widely investigated. This is a major deficiency in the literature, as it makes clinical decision making more difficult when deciding on how to deliver these principles for individual patients.

The division of FRIs into ‘early’ (presenting within 2 weeks of fracture), ‘delayed’ (presenting 3–10 weeks after fracture) and ‘late’ (presenting after 10 weeks) has been advocated to aid decision making in surgery [10,11]. This view is based on the biofilm model of implant infection [12], where the maturation of the biofilm, over time, reduces bacterial susceptibility to systemic anti-microbials [13,14]. However, recent studies have questioned if these rather arbitrary periods are relevant [15]. In a systematic review of FRIs treated with debridement, anti-microbials and implant retention (DAIR), outcomes were not significantly different between one and ten weeks after injury [16].

The use of DAIR, exchange of implants or conversion from internal to external fixation have all been studied separately, but there are few comparisons of the outcome of each strategy [8,11,16,17,18]. There is an agreement that fracture stability is essential in the prevention and treatment of FRIs [9,17,19]. Similarly, the restoration of the soft tissue envelope is recommended [8,18,20,21]. Antimicrobial therapy should be delivered systemically [22], but there is also increasing interest in local anti-microbial delivery in FRIs [9,11,14,23]. The recent guidance issued by the international FRI consensus group [24] highlighted that, although local antibiotics were established in the treatment of prosthetic joint infection, much less data are available on fracture-related infection.

This study evaluated the effect of individual components of care on the outcome of a large consecutive group of patients treated surgically for FRI. We specifically studied the contribution of the presenting features of FRIs, the time from injury, method of stabilization, use of local antibiotics and soft tissue management on the eradication of infection and fracture healing. This observational study allowed for an analysis of these elements together, in a way which has not previously been reported.

## 2. Results

### 2.1. Patient Demographics

A total of 453 patients was eligible for enrollment, but 20 were excluded, as they did not meet the strict inclusion criteria [25]. In total, 433 FRIs were treated in 429 patients with a mean age of 49.7 years (range 17–84). Before surgery, 233 fractures were not healed (53.8%). FRI affected the tibia in 226 (52.2%), the femur in 94 (21.7%), the pelvis in 26 (6%), the radius/ulna in 26 (6%), the humerus in 20 (4.6%) and foot bones in 19 (4.4%) (Table 1). Internal fixation was present in 291 cases (67.2%).

*Staphylococcus aureus* was the most common pathogen (45.5%), with Gram-negative bacteria in 25.9%, coagulase-negative staphylococci in 18.7%, *Streptococcus* spp. in 9.6% and enterococci in 10.4%. Infection was polymicrobial in 35.6% of patients and culture-negative in 14.1%.

Patients were followed up with for a minimum of 12 months (mean 26 months; range 12–72). There were eight deaths during the follow-up, unrelated to the surgery or infection.

### 2.2. Treatment Strategies

When the internal fixation was stable, it was debrided and retained in 140 cases (140/291; 48.1%) where the fracture was not healed. An unstable fixation was removed in 92 unhealed cases (92/291; 31.6%), with the application of an external fixator in 68 and implantation of a new internal fixation in 24. In the remaining 59 cases (59/291; 20.3%), a fixation was removed from a healed fracture.

Direct skin closure was possible in 300 cases (69.3%), with soft tissue reconstruction required in 133 (47 local flaps, 77 free flaps and 9 skin grafts). Negative-pressure wound therapy (NPWT) was used in 64 (14.8%) patients prior to definitive closure.

A total of 251 patients received local implantation of an antibiotic carrier (213 bioabsorbable and 38 non-bioabsorbable PMMA cement). Gentamicin alone was the most frequently used local antibiotic (225/251; 89.3%). Table 2 summarizes the treatment strategies by outcome.

### 2.3. Outcomes

Overall, the eradication of infection was successful in 374/433 FRIs (86.4%), and 209/243 of unhealed infected fractures were healed at the final review (86.0%). In total, 3.3% (9/433) required amputation, equally divided between those with infection recurrence (*n* = 5) or eradication (*n* = 4).

#### 2.3.1. Patient-Related Factors

Having an unhealed fracture at the time of FRI surgery was associated with a higher risk of treatment failure (hazard ratio (HR) 1.939; 95% C.I. 1.075–3.499).

**Figure 1:**
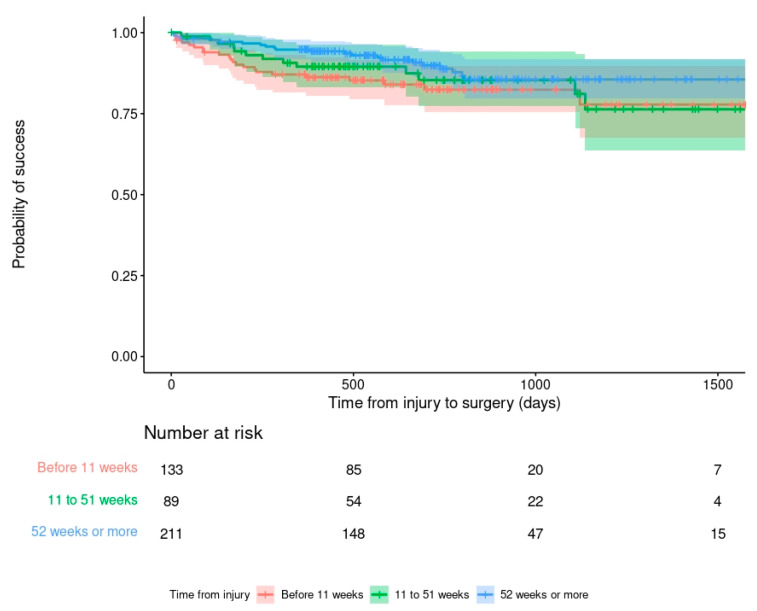
Kaplan–Meier survivorship curve demonstrating the relationship between time from injury to FRI surgery and the primary outcome.

Outcome was not dependent on age, BMI or time from injury (recurrence rate 16.5% in FRI treated at 1–10 weeks after injury; 13.1% at 11–52 weeks; 12.1% at >52 weeks: *p* = 0.52) (Figure 1). Tobacco smoking at the time of surgery increased the chance of failure by three times (HR 3.118; 95% C.I. 1.771–5.491).

The presence of an internal fixation at presentation did not adversely affect the outcome, compared to FRIs without implants (HR 1.583; 95% C.I. 0.82–3.059).

#### 2.3.2. Surgery-Related Factors

**Figure 2:**
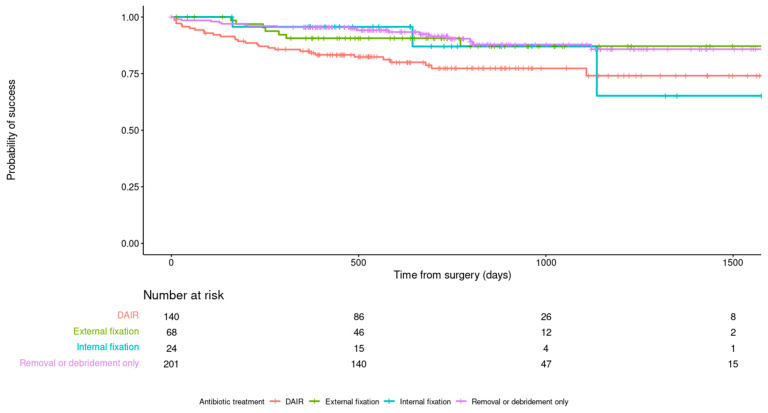
Kaplan–Meier survivorship curve demonstrating the relationship between method of fixation after debridement and primary outcome.

**Figure 3:**
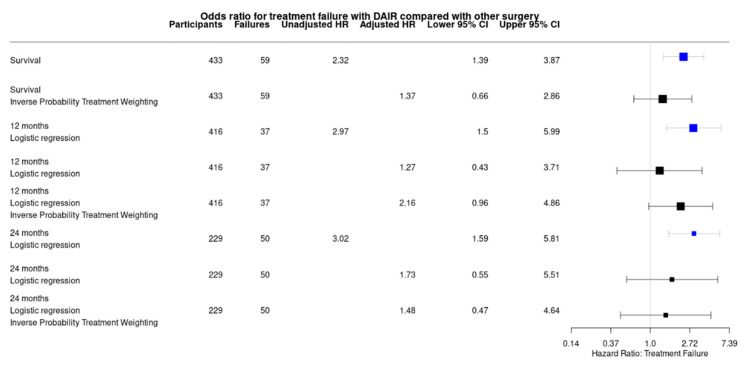
Hazard risk ratios comparing DAIR to other forms of surgery, depending on analysis performed. Point estimates were given with their 95% confidence limits.

**Figure 4:**
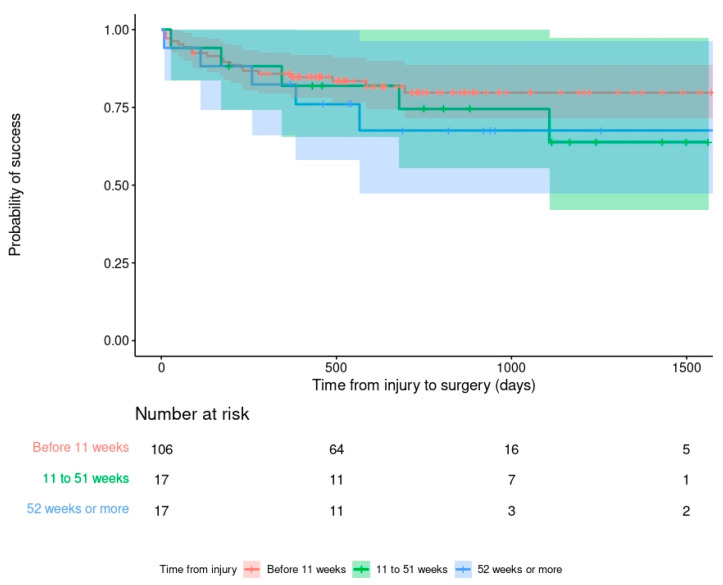
Kaplan–Meier survivorship curve demonstrating the relationship between time from injury to FRI surgery and primary outcome in patients having debridement, anti-microbial therapy and implant retention (DAIR).

The debridement and retention of a stable infected implant (DAIR) had a failure rate of 21.4% (30/140), implant exchange (to a new internal fixation) had a failure rate of 12.5% (3/24) and conversion to an external fixation had a failure rate of 10.3% (7/68) (Figure 2). DAIR was significantly worse than other surgical strategies at 12 and 24 months after treatment in all analyses (Figure 3). Specifically, DAIR was less successful than conversion to an external fixation after the removal of an unstable implant (Adjusted hazard ratio (aHR) 2.377; 95% C.I. 0.986–5.731). There was no effect of the time from injury on the outcome of DAIR (Figure 4) or any other fixation method. However, the number of cases treated with a new internal fixation was relatively small.

Where direct skin closure was possible (*n* = 300), infection was eradicated in 259 cases (86.3%). Soft tissue reconstruction with local or free flaps eradicated infection in 110/124 cases where closure was not possible (88.7%), in all bones. A free tissue transfer in the tibia reduced the failure rate from 19.6% to 7.9% when compared to tibias closed without a free flap (HR 0.38; 95% C.I. 0.14–1.0). NPWT, used prior to any method of skin closure, was associated with a higher rate of treatment failure (HR 3.473; 95% C.I. 1.852–6.512) (Table 2).

A recurrence of infection occurred in 25/251 (10.0%) of patients who received local antibiotic therapy and in 34/182 (18.7%) of those who did not (HR 0.48; 95% C.I. 0.29–0.81: aHR 0.57; 95% C.I. 0.27–1.2). Generally, patients who were treated with local antibiotics had a longer interval from injury to FRI surgery (median 125 weeks; range: 1–3432 vs. median 6.5 weeks; range: 1–1560; *p* < 0.001), but time from injury was not an independent determinant of recurrence in the multi-variate analysis.

## 3. Discussion

This was one of the first large studies to consider outcomes for all patients having all types of surgical treatments for FRI. This pragmatic study design reflects the range of patients seen in daily practice. We included patients treated at three centers, in two countries, to give a broad representation of the range of treatments used in different healthcare settings. Most previous studies reported the results of a specific surgical treatment, such as debridement and implant retention [11,26], antibiotic-coated nailing [27,28], soft tissue reconstruction [20,21] or the use of local antibiotics [23]. Chadayammuri et al. [29] reported on outcomes of 142 infected fractures, but this cohort was under-powered and could not show significant differences in many aspects of care. They also had a high failure rate of over 34% at one year (compared to 13.6% in our cohort). They did, however, show that appropriate systemic anti-microbial therapy was beneficial and reduced the failure rate.

Research into FRIs has been hampered by the lack of an accepted definition of an infected fracture and a working definition of success or failure [30]. The introduction of the FRI consensus definition [7,25] has allowed a standardized approach, which should allow for a comparison between studies in the future. We would advocate the use of the FRI consensus definition in everyday clinical practice. It allowed us to define confirmed infected cases, even when culture-negative and in the absence of open draining wounds.

In this study, we also used the FRI consensus definition to determine the success or failure of our treatments. We postulated that if the confirmatory criteria for FRI were fulfilled at any time after completion of the infection treatment, this would constitute a failure, as the initial indication for treatment was still present. This worked well as the definition, was easy to apply and provided an objective endpoint for treatment failure.

There are many factors which can determine the outcome for patients with FRI [31]. Some of these may be modifiable prior to surgery [32,33]. Our results suggested that smoking cessation may be helpful, particularly if there is no urgency for treatment.

It has traditionally been taught that the treatment of infection early after injury has a better chance of success than with delayed or late-presenting cases. This especially affected our decision making around the use of the debridement and retention of implants (DAIR) in later cases [15,16,26]. In both FRI and PJI, many workers have recommended that DAIR should be reserved for early infections. In this study, we showed that while the use of DAIR was associated with an overall lower success rate, compared to implant removal or conversion to an external fixation, we were not able to show any effect of the time interval from injury on the outcome of DAIR, or indeed, any method of stabilization. This would suggest that time from injury alone should not be used as a criterion for choosing the operative strategy.

Clearly, the outcome of DAIR is dependent on other factors, and our study suggested that the soft tissue cover around the tibia and the use of local antibiotics may also affect this outcome. With modern treatment techniques and careful attention to the principles of care, good results may be obtained with DAIR, at all time points. It has recently been shown that the microbiology of FRIs is not distinctly different when comparing early, delayed or late infections [34]. Hence, the outcome of DAIR cannot be affected by microbiological factors or antibiotic choice.

These results do not suggest that the use of DAIR is always a bad treatment option. A total of 78.6% of cases treated with DAIR was successful and this strategy may be more acceptable to patients. Conversion to an external fixation did have a better outcome (89.7% success), but it can be very difficult in some anatomic locations and inappropriate in some patients. Moreover, in contrast to DAIR performed for a prosthetic joint infection, it is always possible to remove the implant after fracture healing and address the residual infection at that stage. In this series, treating the FRI in a healed bone with the removal of the implant had a high success rate (90.5%).

We would recommend that if DAIR is chosen as a surgical strategy, a careful follow-up of the patient at short, regular intervals is important. The early radiographic loosening/breakage of the implant or skin breakdown would indicate a failure of the treatment. A change of plan would then be required to ensure a better outcome.

Achieving early soft tissue cover has been strongly advocated in the management of open fractures, in order to prevent infection and non-union [5,8,21,35]. In this study, 300 cases could be closed directly with a high success rate (86.3%). A total of 133 cases displayed more severe soft tissue compromise, but the addition of a closure with a local or free tissue transfer allowed a similar high success rate (88.7%) in all bones. In our tibial FRI cases, the addition of a free flap improved the rate of both the fracture union and infection eradication (92.1%).

It can be difficult to assess the needs of soft tissues around infected fractures. In Some cases, which can be closed directly, but with some difficulty, may be better managed with the import of healthy vascularized tissue (local or free flaps) to improve infection eradication and healing. We would postulate that some of the failures in our directly closed cases may have been avoided with the planned use of plastic surgical techniques, particularly around the tibia. This approach requires a considerable organizational infrastructure with combined orthoplastic support [20,21].

Sixty-four patients had NPWT applied after the debridement. The 2018 ICM trauma group [8] recommended that NPWT should be used for less than seven days. The FLOW trial of open tibial fractures suggested that the use of NPWT increased the rate of late infection regardless of the severity of the injury [36,37]. We would agree that it is associated with higher failure rates (HR 3.473; 95% C.I. 1.852–6.512) in the treatment of FRI, but the causal relationship may be complex. Nevertheless, restoring a healthy soft tissue envelope as soon as possible gave the best outcomes in our patients. We recommend that NPWT should not be the preferred method of wound management after debridement. It should only be used for a brief period (definitely less than 7 days), if it is not possible to close the wound at the initial procedure.

The use of local antibiotics has been advocated in the prevention of infection in primary joint replacement, the treatment of prosthetic joint infection and osteomyelitis [24,38,39]. A systematic review of preventative local anti-microbials in open fractures showed a significant benefit, with a three-fold reduction in FRIs in over 2500 patients [40]. We showed a clinical benefit in the treatment of established FRI, when, combined with a good debridement, systemic anti-microbials, stabilization and soft tissue cover, it reduced failure rates by half. Recently introduced bioabsorbable antibiotic carriers can fill small bone defects and cover retained metalwork. They provide high concentrations of anti-microbials directly around the fracture, without concerns about tissue perfusion or patient compliance, which reduce the effectiveness of systemic antibiotics.

This study was limited by the heterogeneity of the cases, but this reflected the range of patients who presented with FRI. By using multiple analysis techniques, we were able to study the important contributors to treatment (Figure 2), but this provided only an overview of the issue. We were not able to comment on the mechanisms by which each element of treatment affected the outcome. Our results indicated areas which would benefit from further study.

There would also be variability in the experience of surgeons providing treatment. We were not able to study this specifically and acknowledge that this would contribute to the effectiveness of any procedure.

## 4. Materials and Methods

All adult patients presenting with possible fracture-related infection between January 2015 and December 2019 at the Bone Infection Unit, Oxford University Hospitals, UK, and the Departments of Trauma Surgery, University Medical Centers Utrecht and Groningen, the Netherlands, were eligible for recruitment. Patients were included if they had surgery for FRI, confirmed using the updated international consensus definition of FRIs [7,25]. All infected fractures of the appendicular skeleton and pelvis were included. Fractures of the skull/facial bones, spine, hand and pathological fractures were excluded. Patients were excluded if less than 3 deep tissue specimens were taken for microbiological culture at surgery, or if optimal definitive surgery for FRI was not performed (due to conservative management, patients with polytrauma preventing optimal surgery, patients declining optimal surgery and surgery not being possible due to logistical issues) (*n* = 20). Patients were followed up for a minimum of one year or until death or amputation (if less than one year).

The diagnosis, type of surgery and anti-microbial therapy were determined by a multi-disciplinary team, comprising infectious disease physicians; trauma, orthopedic and plastic surgeons; radiologists, microbiologists and clinical pharmacists. There was no restriction on the time from injury to FRI surgery, the type of surgery or soft tissue reconstruction.

Data were retrieved from prospectively collected databases and retrospective data from review of medical records and microbiology laboratory databases. Patient-related factors (age, BMI, co-morbidities, anatomical site of the FRI, degree of healing of the fracture and clinical features) were recorded together with details of the surgical treatment (time from injury to FRI surgery, method of stabilization, need for soft tissue reconstruction, use of local antibiotics and use of negative-pressure wound therapy). Clinical, microbiological and histological data were collected to confirm the presence of FRI [25] and to identify the pathogens requiring anti-microbial treatment.

All patients received intravenous empiric anti-microbial therapy, administered according to the local epidemiology and resistance patterns of participating hospitals and included both Gram-positive and Gram-negative coverage. This was followed by culture-specific antibiotics after the results of intraoperative specimens were obtained.

The primary outcome measure was the rate of eradication of the infection. Failure was defined by (i) the presence of any confirmatory signs of FRI [7,25], (ii) the use of new anti-microbials to treat infective symptoms (other than a short course oral therapy for an external fixator pin tract infection) or (iii) an unplanned surgery for a possible infection, at any time point between the completion of the initial FRI surgery and the final follow-up. The outcome was assessed, blinded to treatment details, at a minimum of one year by three independent reviewers.

Secondary analysis defined the rate of union in unhealed FRIs and the amputation rate. The effectiveness of individual components of treatment on final outcome were then compared. Univariate and multi-variate analyses were performed using Chi square tests, adjusted and unadjusted Cox proportional hazards modelling and logistic regression for infection recurrence. Missing baseline variables (<3%) were imputed using multiple imputation with chained equations (MICE). Inverse probability of treatment weighting (IPTW) was used to account for confounding in the causal pathway between surgical treatment and infection recurrence. Deaths and amputations were taken as censor events, as they prevented the subsequent diagnosis of infection recurrence.

## 5. Conclusions

Successful outcomes in FRIs are multi-factorial and require careful attention to the principles of care, preferably delivered by a multi-disciplinary team. Using the new FRI consensus definition, we were able to study a large group of patients with confirmed infection and explore the contributions of several elements of care on the outcomes.

Our data showed that the debridement and retention of stable implants had a higher risk of failure (HR 2.377) but may still be an appropriate treatment for many patients (successful in 78.6%). NPWT was associated with an increase in infection recurrence (HR 3.473), while local antibiotics reduced recurrence from 18.7% to 10.0%.

The treatment strategy must be tailored to the individual needs of the patient. We provided new outcome data, which could inform decision making together with patients. Overall, the results were good, but there is room for improvement. Focus on smoking cessation, defining better indications for DAIR, limiting NPWT use and the improved use of flap reconstruction and local antibiotics are all areas which offer the potential for improved outcome. Interestingly, outcomes were not affected by the time from injury to FRI surgery, suggesting that the division of FRIs into categories based on the time from injury may not be helpful with modern treatment algorithms.

## Figures and Tables

**Table 1:** Patient demographics related to treatment outcome.

Variable		Treatment Success	Treatment Failure
		(*n* = 374)	(*n* = 59)
Age (mean, range)		49.8 (18 to 84)	48.2 (20 to 74)
Gender	Male	270	48
	Female	104	11
BMI		27.5 (12.5 to 47.0)	27.5 (18.5 to 40.8)
Current smoking	Yes	84 (22.5%)	28 (47.5%)
	No	290	31
Diabetes mellitus		39 (10.4%)	8 (13.6%)
Immunosuppressant use		14 (3.7%)	3 (5.4%)
Time from injury to surgery in weeks		33.8 (0 to 3432)	23.3 (1 to 1820)
Mean (range) median		48	35
Diagnostic criteria	Sinus or fistula	199 (53.2%)	25 (42.4%)
	Purulence in fracture site	138 (37.0%)	25 (42.4%)
	Microbiology ≥2 specimens culture-positive	282 (75.4%)	51 (86.4%)
	Microbiology 1 specimen positive	36 (9.6%)	3 (5.1%)
	Histopathology positive	157 (42.0%)	14 (23.7%)
	Histopathology negative	68 (18.2%)	3 (5.4%)
	Pre-operative CRP (mean, range)	53.6 (0 to 480)	57.4 (1 to 274)
Bone	Tibia	189	37
	Fibula	12	1
	Foot	13	6
	Femur	81	13
	Pelvis	26	0
	Clavicle	6	0
	Humerus	20	0
	Radius/ulna	24	2
	Other	3	0
Fracture healed?	Yes	172 (46.0%)	18 (30.5%)
	No	202 (54.0%)	41 (69.5%)
Pre-operative fixation?	None	122 (32.6%)	13 (22.0%)
	Internal	249 (66.6%)	42 (71.1%)
	External	3 (0.8%)	4 (6.8%)
Followed-up (survivors *)	At least 12 months	363/368 (98.6%)	58/58 (100%)
	At least 24 months	170/363 (46.8%)	38/58 (65.5%)
Amputation or mortality during follow-up		4 amputations 7 deaths	5 amputations 1 death

* Includes all patients reviewed who did not die or had an amputation before 12 or 24 months.

**Table 2:** Treatment strategy related to treatment outcome.

Variable		Treatment Success	Treatment Failure
		(*n* = 374)	(*n* = 59)
Surgical approach	Debridement +/− implant removal	182 (48.7%)	19 (32.2%)
	External fixation	61 (16.3%)	7 (11.9%)
	Internal fixation	21 (5.6%)	3 (5.1%)
	DAIR	110 (29.4%)	30 (50.8%)
Closure and plastic surgical approach	Direct closure	259 (69.2%)	41 (69.5%)
	Split skin graft only	5 (1.3%)	4 (6.8%)
	Local flap	39 (10.4%)	8 (13.6%)
	Free flap	71 (19.0%)	6 (10.2%)
NPWT used prior to skin closure	Yes	45 (12%)	19 (32.2%)
	No	329 (88%)	40 (67.8%)
Local anti-microbial therapy	Yes	227 (60.7%)	25 (42.4%)
	No	147 (39.3%)	34 (57.6%)

DAIR: debridement, anti-microbial therapy and implant retention; NPWT: negative-pressure wound therapy.

## References

[1] Baertl S., Metsemakers W.J., Morgenstern M., Alt V., Richards R.G., Moriarty T.F., Young K. (2021). Fracture-related Infection. Bone Jt. Res..

[2] Alt V., Giannoudis P.V. (2019). Musculoskeletal infections– A global burden and a new subsection in Injury. Injury.

[3] Iliaens J., Onsea J., Hoekstra H., Nijs S., Peetermans W.E., Metsemakers W.-J. (2021). Fracture-related infection in long bone fractures: A comprehensive analysis of the economic impact and influence on quality of life. Injury.

[4] Olesen U.K., Pedersen N.J., Eckardt H., Lykke-Meyer L., Bonde C.T., Singh U.M., McNally M. (2017). The cost of infection in severe open tibial fractures treated with a free flap. Int. Orthop..

[5] Metsemakers W.-J., Onsea J., Neutjens E., Steffens E., Schuermans A., McNally M., Nijs S. (2017). Prevention of fracture-related infection: A multidisciplinary care package. Int. Orthop..

[6] Hak D.J., Fitzpatrick D., Bishop J.A., Marsh J.L., Tilp S., Schnettler R., Simpson H., Alt V. (2014). Delayed union and nonunions: Epidemiology, clinical issues and financial aspects. Injury.

[7] Metsemakers W.J., Morgenstern M., McNally M.A., Moriarty T.F., McFadyen I., Scarborough M., Athanasou N.A., Ochsner P.E., Kuehl R., Raschke M. (2018). Fracture-related infection: A consensus on definition from an international expert group. Injury.

[8] Obremskey W.T., Metsemakers W.-J., Schlatterer D.R., Tetsworth K., Egol K., Kates S., McNally M. (2020). Musculoskeletal Infection in Orthopaedic Trauma. Assessment of the 2018 International Consensus Meeting on Musculoskeletal Infection. J. Bone Jt. Surg. Am..

[9] McNally M.A., Kates S.L., Borens O. (2016). Infection after Fracture. Principles of Orthopedic Infection Management.

[10] Willenegger H., Roth B. (1986). Treatment tactics and late results in early infection following osteosynthesis. Unfallchirurgie.

[11] Sendi P., Morgenstern M., Metsemakers W.-J., McNally M.A., Zimmerli W. (2021). Fracture-related Infection of long bones. Bone and Joint Infections: From Microbiology to Diagnostics and Treatment.

[12] Gristina A.G., Costerton J.W. (1984). Bacterial adherence and the glycocalyx and their role in musculoskeletal infection. Orthop. Clin. N. Am..

[13] Stewart P.S., Costerton J.W. (2001). Antibiotic resistance of bacteria in biofilms. Lancet.

[14] Masters E.A., Trombetta R.P., de Mesy Bentley K.L., Boyce B.F., Gill A.L., Gill S.R., Nishitani K., Ishikawa M., Morita Y., Ito H. (2019). Evolving concepts in bone infection: Redefining “biofilm”, “acute vs. chronic osteomyelitis”, “the immune proteome” and “local antibiotic therapy”. Bone Res..

[15] Aytaç S., Schnetzke M., Swartman B., Herrmann P., Woelfl C., Heppert V., Gruetzner P.A., Guehring T. (2014). Posttraumatic and postoperative osteomyelitis: Surgical revision strategy with persisting fistula. Arch. Orthop. Trauma. Surg..

[16] Morgenstern M., Kuehl R., Zalavras C.G., McNally M., Zimmerli W., Burch M.A., Vandendriessche T., Obremskey W.T., Verhofstad M.H.J., Metsemakers W.J. (2021). The influence of duration of infection on outcome of Debridement and Implant Retention (DAIR) in Fracture-related Infection; A systematic review and critical appraisal. Bone Jt. J..

[17] Metsemakers W.-J., Morgenstern M., Senneville E., Borens O., Govaert G.A.M., Onsea J., Depypere M., Richards R.G., Trampuz A., On behalf of the Fracture-Related Infection (FRI) group (2020). General treatment principles for fracture-related infection: Recommendations from an international expert group. Arch. Orthop. Trauma. Surg..

[18] Bezstarosti H., Van Lieshout E.M.M., Voskamp L.W., Kortram K., Obremskey W., McNally M.A., Metsemakers W.J., Verhofstad M.H.J. (2019). Insights into treatment and outcome of fracture-related infection: A systematic literature review. Arch. Orthop. Trauma. Surg..

[19] Foster A.L., Moriarty T.F., Zalavras C., Morgenstern M., Jaiprakash A., Crawford R., Burch M.-A., Boot W., Tetsworth K., Miclau T. (2020). The influence of biomechanical stability on bone healing and fracture-related infection: The legacy of Stephan Perren. Injury.

[20] Chan J., Ferguson J., Scarborough M., McNally M.A., Ramsden A. (2019). Management of Post-traumatic Osteomyelitis in the Lower Limb: Current State of the Art. Indian J. Plast. Surg..

[21] Müller S.L., Morgenstern M., Kuehl R., Muri T., Kalbermatten D.F., Clauss M., Schaefer D.J., Sendi P., Osinga R. (2021). Soft tissue reconstruction in lower leg fracture-related infections: An orthoplastic outcome and risk factor analysis. Injury.

[22] Depypere M., Kuehl R., Metsemakers W.J., Senneville E., McNally M.A., Obremskey W.T., Zimmerli W., Atkins B.L., Trampuz A. (2020). Recommendations for systemic antimicrobial therapy in fracture-related infection: A consensus from an international expert group. J. Orthop. Trauma.

[23] Pesch S., Hanschen M., Greve F., Zyskowski M., Seidl F., Kirchhoff C., Biberthaler P., Huber-Wagner S. (2020). Treatment of fracture-related infection of the lower extremity with antibiotic-eluting ceramic bone substitutes: Case series of 35 patients and literature review. Infection.

[24] Metsemakers W.-J., Fragomen A.T., Moriarty T.F., Morgenstern M., Egol K., Zalavras C., Obremskey W.T., Raschke M., McNally M.A. (2019). Evidence-based recommendations for local antimicrobial strategies and dead space management in Fracture-related Infection. J. Orthop. Trauma.

[25] McNally M., Govaert G., Dudareva M., Morgenstern M., Metsemakers W.-J. (2020). Definition and diagnosis of fracture-related infection. EFORT Open Rev..

[26] Kuehl R., Sutter S.T., Morgenstern M., Dangel M., Egli A., Nowakowski A., Suhm N., Theilacker C., Widmer A.F. (2019). Time-dependent differences in management and microbiology of orthopaedic internal fixation-associated infections: An observational prospective study with 229 patients. Clin. Microbiol. Infect..

[27] Conway J., Mansour J., Kotze K., Specht S., Shabtai L. (2014). Antibiotic cement-coated rods: An effective treatment for infected long bones and prosthetic joint nonunions. Bone Jt. J..

[28] Makhdom A.M., Buksbaum J., Rozbruch S.R., Da Cunha R., Fragomen A.T. (2020). Antibiotic Cement-Coated interlocking Intramedullary Nails in the Treatment of Septic Complex Lower Extremity Reconstruction; A Retrospective Analysis with Two year Minimum Follow Up. J. Bone Jt. Infect..

[29] Chadayammuri V., Herbert B., Hao J., Mavrogenis A., Quispe J.C., Kim J.W., Young H., Hake M., Mauffrey C. (2017). Factors associated with adverse postoperative outcomes in patients with long bone post-traumatic osteomyelitis. Eur. J. Orthop. Surg. Traumatol..

[30] Metsemakers W.J., Kortram K., Morgenstern M., Moriarty T.F., Meex I., Kuehl R., Nijs S., Richards R.G., Raschke M., Borens O. (2018). Definition of infection after fracture fixation: A systematic review of randomized controlled trials to evaluate current practice. Injury.

[31] Metsemakers W.J., Kuehl R., Moriarty T.F., Richards R.G., Verhofstad M.H., Borens O., Kates S., Morgenstern M. (2018). Infection after fracture fixation: Current surgical and microbiological concepts. Injury.

[32] Hotchen A.J., Dudareva M., Corrigan R.A., Ferguson J.Y., McNally M.A. (2020). Can we predict outcome after treatment of long bone osteomyelitis? A study of patient-reported quality of life, stratified with the BACH Classification. Bone Jt. J..

[33] Dudareva M., Hotchen A., McNally M.A., Hartmann-Boyce J., Scarborough M., Collins G. (2021). Systematic review of risk prediction studies in bone and joint infection: Are modifiable prognostic factors useful in predicting recurrence?. J. Bone Jt. Infect..

[34] Walter N., Baertl S., Engelstaedter U., Ehrenschwender M., Hitzenbichler F., Alt V., Rupp M. (2021). Letter in response to article in journal of infection: “The microbiology of chronic osteomyeleitis: Changes over ten years”. J. Infect..

[35] British orthopaedic Association Trauma Committee (2020). BOA Standard for Trauma: Open frature management. Injury.

[36] The FLOW Investigators (2015). A trial of wound irrigation in the initial management of open fracture wounds. N. Engl. J. Med..

[37] Sendi P., McNally M.A. (2016). Wound irrigation in initial management of open fractures. N. Engl. J. Med..

[38] Mifsud M., McNally M.A. (2019). Local delivery of antimicrobials in the treatment of bone infections. Orthop. Trauma.

[39] Ferguson J., Diefenbeck M., McNally M. (2017). Ceramic biocomposites as biodegradable antibiotic carriers in the treatment of bone infections. J. Bone Jt. Infect..

[40] Morgenstern M., Vallejo A., McNally M., Moriarty F., Ferguson J., Nijs S., Metsemakers W. (2018). The effect of local antibiotic prophylaxis in open limb fractures: A systematic review and meta-analysis. Bone Jt. Res..

